# A Precisely Regulated Gene Expression Cassette Potently Modulates Metastasis and Survival in Multiple Solid Cancers

**DOI:** 10.1371/journal.pgen.1000129

**Published:** 2008-07-18

**Authors:** Kun Yu, Kumaresan Ganesan, Lay Keng Tan, Mirtha Laban, Jeanie Wu, Xiao Dong Zhao, Hongmin Li, Carol Ho Wing Leung, Yansong Zhu, Chia Lin Wei, Shing Chuan Hooi, Lance Miller, Patrick Tan

**Affiliations:** 1 National Cancer Centre, Singapore, Singapore; 2 Department of Physiology, National University of Singapore, Singapore, Singapore; 3 Duke–NUS Graduate Medical School, Singapore, Singapore; 4 Genome Institute of Singapore, Singapore, Singapore; Stanford, United States of America

## Abstract

Successful tumor development and progression involves the complex interplay of both pro- and anti-oncogenic signaling pathways. Genetic components balancing these opposing activities are likely to require tight regulation, because even subtle alterations in their expression may disrupt this balance with major consequences for various cancer-associated phenotypes. Here, we describe a cassette of cancer-specific genes exhibiting precise transcriptional control in solid tumors. Mining a database of tumor gene expression profiles from six different tissues, we identified 48 genes exhibiting highly restricted levels of gene expression variation in tumors (n = 270) compared to nonmalignant tissues (n = 71). Comprising genes linked to multiple cancer-related pathways, the restricted expression of this “Poised Gene Cassette” (PGC) was robustly validated across 11 independent cohorts of ∼1,300 samples from multiple cancer types. In three separate experimental models, subtle alterations in PGC expression were consistently associated with significant differences in metastatic and invasive potential. We functionally confirmed this association in siRNA knockdown experiments of five PGC genes (*p53CSV*, *MAP3K11*, *MTCH2*, *CPSF6*, and *SKIP*), which either directly enhanced the invasive capacities or inhibited the proliferation of AGS cancer cells. In primary tumors, similar subtle alterations in PGC expression were also repeatedly associated with clinical outcome in multiple cohorts. Taken collectively, these findings support the existence of a common set of precisely controlled genes in solid tumors. Since inducing small activity changes in these genes may prove sufficient to potently influence various tumor phenotypes such as metastasis, targeting such precisely regulated genes may represent a promising avenue for novel anti-cancer therapies.

## Author Summary

Successful carcinogenesis involves the integration of both pro- and anti-oncogenic pathways. We postulated that genes critical for balancing these opposing pathways are likely to be precisely controlled in tumors, since even subtle alterations in their activity might cause substantial alterations in tumor growth and survival. Using a novel genomic approach, we identified a 48-gene “Poised Gene Cassette” (PGC) showing tight regulation specifically in human cancers but not in corresponding nonmalignant tissues. We show, using a wide variety of *in vitro* and *in vivo* approaches, that small alterations in PGC expression are consistently associated with significant differences in experimental metastasis and patient survival, and we demonstrate a direct functional role for five PGC genes (*p53CSV*, *MAP3K11*, *MTCH2*, *CPSF6* and *SKIP*) in cancer invasion. Our findings support the existence of a novel class of ultrasensitive genes that may regulate various cancer-associated phenotypes such as metastasis. Such precisely controlled genes could represent appealing drug targets, since even partial alterations in their activity should prove sufficient to induce potent effects on tumors. Besides cancer, our analytical approach is quite generalizable and likely to be applicable to other disease conditions.

## Introduction

The accurate processing and integration of multiple external signals is a common feature of biological networks in normal health and complex disease. As illustrated by the examples of oxygen handling [1], energy control [2], and ion homeostasis [3], such accuracy frequently involves the precise coordination of multiple cellular pathways, and mechanisms for regulating and balancing opposing activities. In cancer networks, many similar requirements for pathway balance are likewise found as successful tumorigenesis requires the robust integration of both pro- and anti-oncogenic pathways controlling cellular proliferation, apoptosis, motility, adhesion and senescence [4],[5]. The importance of balancing opposing activities in cancer is illustrated by genes such as *HEF1* (*NEDD9*), a metastasis-related gene [6] and *HMMR*, a gene involved in centrosome formation (Pujana et al. 2007). Either repression or overexpression of *HEF1* can cause mitotic defects [7],[8], indicating that its activity in tumors requires tight regulation. Similarly, subtle alterations of *HMMR* expression in normal mammary tissues may promote breast tumorigenesis, underscoring the need to keep the *HMMR* gene tightly regulated [9]. Such findings support the notion that balancing the activity of positive and negative effectors is likely to be a central requirement of many cancers.

At the systems-level, pathway balance is often facilitated through the use of network structures [10] conveying robustness to random fluctuations and errors [11]–[14]. However, the pivotal balancing role played by certain genetic components may at least partially explain why some networks also exhibit ultrasensitivity – a phenomena where small changes in activity at specific components can suffice to elicit qualitative changes in output [12],[13]. Ultrasensitivity may contribute to a network's ability to rapidly respond to changing environmental and genetic conditions [15],[16]. Intriguingly, there is emerging evidence that certain cancers can also display ultrasensitivity. Some remarkable examples include the dramatic responses of chronic lymphocytic leukemia cells to colchicines, occurring at concentrations 10,000-fold lower than that required for similar effects on normal lymphocytes [17],[18], and the striking clinical responses of certain solid tumors to targeted pathway inhibitors [19]. From a therapeutic perspective, such ultrasensitive components could prove particularly appealing as drug targets, as even small alterations might prove sufficient to induce potent effects on tumor phenotypes such as tissue invasion and metastasis. However, our current understanding of the role that ultrasensitivity plays in cancer is still far from complete. Identifying additional genetic components regulating pathway balance in tumors might thus improve our ability to target critical control nodes in cancer networks.

As a general strategy to identify ultrasensitive components in tumors, we hypothesized that a) such components should be precisely regulated and thus exhibit restricted levels of expression variation in cancers; and b) subtle alterations in the expression levels of these components should induce or be associated with significant phenotypic changes. We then applied these criteria to determine if such precisely-regulated genes might be inferred from databases of tumor gene expression profiles. While several groups have compared the expression profiles of multiple tumor and non-malignant tissues [20],[21], to our knowledge, no study to date has systematically attempted to investigate the issue of precise gene regulation in tumors. Employing a genome-wide computational strategy, we identified and robustly validated a novel “Poised Gene Cassette” (PGC) of genes undergoing precise regulation in a microarray database of human tumors from diverse tissue types. Furthermore, subtle alterations in PGC expression were associated with significant and measurable alterations in important tumor phenotypes such as experimental metastasis and patient survival. Our results thus suggest the existence of a generalized homeostatic mechanism in solid tumors for maintaining precise levels of PGC transcription, which may be important for various cancer-associated phenotypes, such as tissue invasion and metastasis. Importantly, the approach described in this study is quite generalizable and can be applied to other diseases.

## Results

### Defining a Cassette of Precisely Regulated Genes in Multiple Solid Tumors

**Figure 1:**
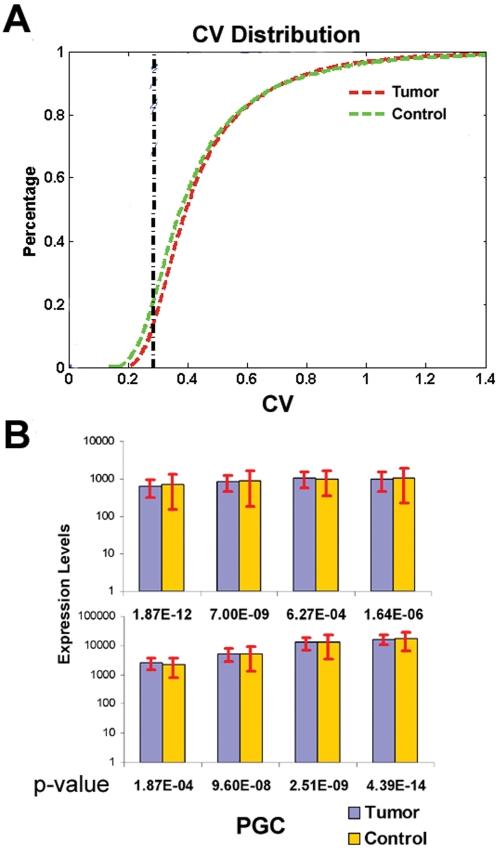
Gene expression variation in tumor and non-malignant samples. A) Distribution of gene expression variation (CV) in tissue samples. Equal numbers of tumor and control samples (50) were randomly selected from the training set to generate a cumulative distribution graph depicting the genome-wide distribution of CVs across tumors (red line) and controls (green line). Genes to the left of the distribution curve correspond to genes with decreased CV (stably expressed), while genes on the right are associated with increased CV. The threshold CV_T_ of 0.28 (black dotted line) represents the CV value where ∼16% of genes in tumor samples are considered to be tightly regulated (i.e., CV<CV_T_, see Main Text for details). B) Expression variation of individual PGC genes in tumors and non-malignant samples. The height of the bar chart represents each gene's mean expression level (in log scale) across all tumor and normal samples in the training set. Error bars (red lines) represent 2 standard deviations of expression values. PGC genes show significantly restricted variation in tumors (blue bar) related to control (yellow bar). P-values calculated by F-test were provided for each PGC gene (x-axis). Note that the mean expression levels of the PGC genes are similar between tumors and controls.

We hypothesized that genes precisely regulated in cancer should exhibit a highly restricted level of gene expression variation across a large database of individual tumor gene expression profiles. To investigate this, we generated gene expression profiles for 270 primary tumors from six tissue types (breast, colon, liver, lung, oesophageal and thyroid) using Affymetrix U133A Genechips. For every gene, we computed gene expression coefficient of variances (CV), where genes with small CVs are considered more tightly regulated than genes with large CVs. We focused on the top 15% most tightly-regulated genes in tumors, corresponding to an empirical CV cut-off of 0.28. To identify genes whose tight regulation was tumor-specific, we used a second database of 71 adjacent matched non-malignant tissues (“control” tissues) to eliminate from this 15% genes that were also tightly regulated in non-malignant samples (CV>0.3). The use of an absolute CV threshold is permissible, as the global distribution of expression CVs between tumors and controls were highly similar (mean CVs were 0.46 and 0.45 for tumors and controls) (Figure 1A). Using this criterion, we identified a “Poised Gene Cassette” (PGC) of 48 genes exhibiting highly restricted levels of expression variation in tumors (Figure 1B). The F-test, a statistical method for comparing the variation of different data sets, confirmed that each of the 48 PGC genes was indeed associated with significantly decreased expression variation in tumors relative to controls (one tailed F-test, p = 0.0001 to 4×10^−14^). We also varied the CV threshold between 0.26–0.3 (+/−7%) and repeated the analysis. Similar results were obtained (Table S1), indicating that the identification of PGC is not dependent on a particular CV threshold.

### The PGC Is not Biased Towards Probe Selection, Normalization Technique, Expression Level, or Sample Selection

We investigated whether the reduced expression variation of the PGC might be due to technical features of the Affymetrix platform or the composition of the initial training set. We considered the possibility that the reduced variance of the PGC might be due to an overabundance of ‘poor quality’ probes, which might be expected to cross hybridize with multiple genes and hence generate higher background signals [22]. However, an examination of a vendor provided list of questionable probes (i.e., with ‘_s_at’ and ‘_x_at’ suffixes), confirmed that the PGC was not significantly enriched in poor quality probes (p = 0.4). In addition, a comparison of the PGC genes against an in-house curated list of unreliable array probes based on sequence redundancy and repeat mapping [23] confirmed that unreliable probes were not overrepresented in the set of PGC genes (p = 0.8).

**Figure 2:**
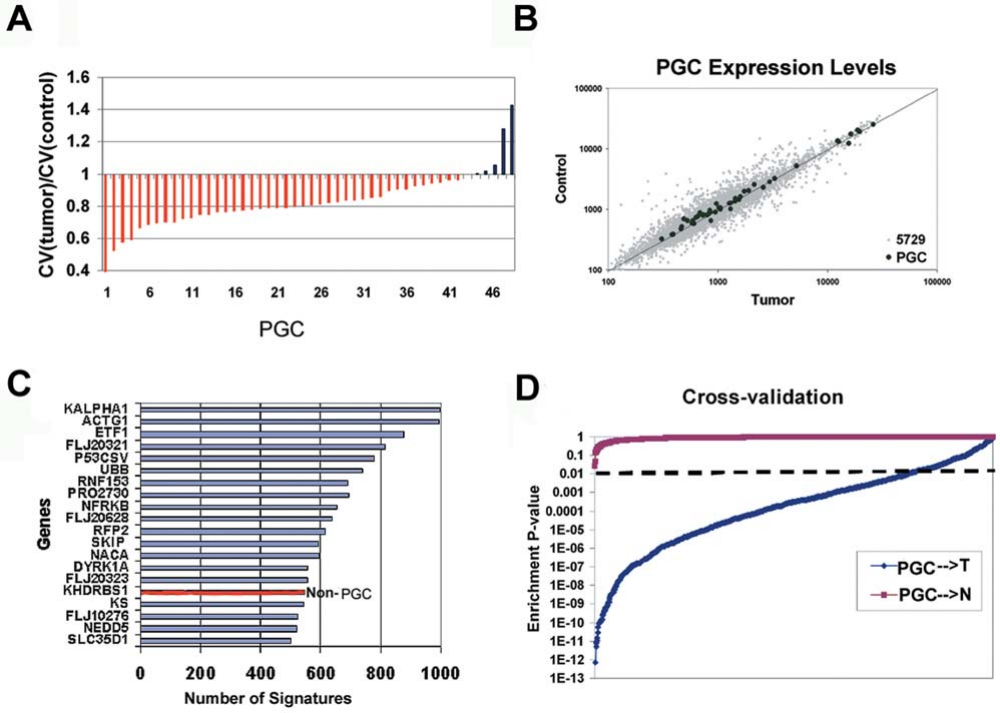
Identification and Cross-validation of the PGC. A) CV of PGC genes in RMA-processed data. Y-axis is the ratio for CV(tumor)/CV(control). Each bar represents a PGC gene. Red bars represent PGC genes with ratio less than 1, indicating the expression variation in tumors is smaller than in controls. Blue bars represent PGC genes with ratio greater than 1. B) Distribution of absolute expression levels for individual PGC genes. For each gene, the X-axis represents its expression level in tumors and the Y-axis its expression in non-malignant tissues. The PGC genes (black spots) located around the diagonal line (i.e., expression ratio of control/tumor = 1) are randomly scattered across the whole 5729-gene set (gray spots). C) and D) Repeated Random Sampling (RRS) to assess PGC robustness. C) Genes belonging to the original PGC signature (blue lines) that were re-identified in at least 500 of 1,000 re-sampled RRS PGC signatures. The red bar represents the only gene (*KHDRBS1*) not belonging to the original PGC signature that was re-selected to the same frequency (i.e. >50%). D) Robustness of re-selected PGC signatures in 1,000 cross-validation test sets. The PGC gene set was queried against either the set of tumor (blue line) or non-malignant tissues (red line) in the cognate RRS cross-validation test set. PGC→T in the figure indicates the PGC→Tumor_test_ comparison. The Y-axis represents the statistical significance of overlap between any re-selected PGC signature and the cohort of tightly regulated genes in the tumor and non-malignant test sets (assessed by the hypergeometric distribution, with p-values of p<0.01 (dotted line) being deemed significant).

To investigate the influence of normalization protocol on PGC discovery, we re-processed the training set using a different normalization method (RMA, [24],[25]). In the RMA-normalized data, we found that 90% of the original PGC genes still exhibited decreased expression variation in tumors relative to controls (i.e., CV(control)>CV(tumors)) (Figure 2A). Thus, the tumor-specific restricted expression variation of the PGC does not appear to be dependent upon a specific normalization technique.

The reduced variation of the PGC is also not due to an overrepresentation of either high-expressing or low-expressing genes. As shown in Figure 2B, the PGC genes were equally distributed across a wide range of expression levels and not confined to either low or highly expressing genes in tumors or control tissues. Thus, the reduced expression variation of the PGC in cancers is unlikely to be due to the PGC genes simply being either highly expressed, rendering the PGC distinct from some studies suggesting an inverse correlation between expression variation and absolute expression levels [26]. Similarly, the PGC is also not biased in lowly expressed genes, consistent with our original selection criteria requiring these genes to be reliably detected in the majority of samples (see Methods). It is also important to note that the PGC genes do not exhibit significant differences in their absolute mean expression levels between cancers and normal tissues (Figures 1B and 2B), but instead only differ in their levels of expression variation between cancers and normal tissues. This observation, as well as others, also provides an argument that the PGC genes are unlikely to represent tissue-specific expression (see Discussion).

The discovery of the PGC is also not influenced by the overrepresentation of breast tumors in our initial training set (breast tumors comprised 68% of the training set). Specifically, we removed all the breast tissues and repeated the PGC analysis. Even without inclusion of breast tissues, 83% (40/48) of the PGC genes still exhibited reduced variation in tumors compared to controls. Of 47 genes exhibiting tumor-specific tight regulation in the breast-excluded data (CV<0.28), 24 genes were part of the original PGC, an overlap far beyond random chance (50%, p = 1.3E-11, hypergeometric test). Taken collectively, these results suggest that the identification of the PGC, and its restricted expression variation in cancers, is unlikely to be due to a technical artifact or the inclusion of a specific cancer type.

### A Cross-Validation Assay Confirms Specificity and Robustness of the PGC Signature

To confirm that the restricted expression variation of the PGC was specifically associated with malignancy, we determined the frequency at which a member gene of the PGC could be re-identified in a series of class-permutation tests. When the class labels of the samples (i.e., tumor or control) were shuffled to generate a series of 1000 permuted sets, almost all the PGC genes (46/48, 96%) could only be re-identified in less than 5% of the class-permuted signatures, consistent with the decreased expression variation of the PGC being tightly associated with tumor samples.

We then evaluated the robustness of the PGC by repeated random sampling (RSS), a stringent cross-validation strategy [27]. The original training set was randomly divided 1000 times into two parts, generating a large series of distinct training/test set combinations. For each of the 1000 derived RSS training sets, we identified new PGC signatures (rPGC) and compared them to the original PGC gene set. Following the guidelines of Michels et al [27], 20 genes were repeatedly selected in more than half of the 1,000 new rPGC signatures. Of these 20 genes, 19 (95%) are members of the original 48-gene PGC (Figure 2C) – the observation that only one gene not part of the original PGC signature was repeatedly selected in the RSS assay indicates that a substantial proportion of the PGC signature (40%) is robust to training set selection. To evaluate the transportability of the PGC signatures, we then applied each of the 1000 rPGC signatures to their cognate test sets. In anticipation that most independent test sets are likely to contain either tumor or control samples but not both, we considered the tumors and controls separately from one another in this analysis. In each test set, we checked if the population of tightly regulated genes, defined using the original CV_T_ threshold (0.28), contained a significant enrichment of rPGC genes (see Methods). The rPGC signatures were significantly enriched in the population of tightly regulated genes in 80% of the tumor test sets (PGC→T, Figure 2D), and importantly were NOT significantly enriched in 100% of the control test sets (PGC→N, Figure 2D), indicating that the PGC is robust in recapitulating its precise regulation in multiple tumor data sets, but not data sets of non-malignant samples. Together, these results confirm the specificity of the PGC for tumors.

### Independent Validation of the PGC in Diverse Solid Tumors

We then asked if the precision of PGC regulation in cancer could be observed in independent data sets of diverse tumors. We collected nine independent cancer cohorts, comprising in total 1105 cancer samples from >7 primary tissue types [28]–[32], including I) four tissues not represented in the original training data (gliomas, gastric, NPC, and ovarian), II) one data set (Yu_Gastric&NPC) representing a mix of two different tissues, and III) a collection of cancer cell lines (NCI60) from nine different tissues. A summary of these nine data sets can be found in Table S2 and the corresponding references. Using a similar strategy to the RSS test sets, a significant fraction of the PGC genes were tightly regulated in all nine primary tumor data sets (p-value range: 0–0.002) (Table 1A), confirming the existence of the PGC in a wide variety of solid tumors. In total, 19 out of 48 PGC genes repeatedly exhibited reduced expression variation in more than half of the 9 cancer test sets (Table S3). We also performed the reciprocal experiment and evaluated the regulation of the PGC in a series of independent non-malignant samples. Although such datasets are rarer in their availability and typically smaller than cancer datasets, we collected two distinct cohorts comprising 115 normal tissues from various organs [33],[34]. Notably, these non-malignant samples were obtained from healthy donors, and are thus free of malignancy and representative of true normal samples. In stark contrast to the cancer data sets, the PGC genes exhibited either no or only a marginal degree of tight regulation in the normal data sets (p = 0.07 and 0.01; Table 1B). Thus, these results indicate that the precise regulation of the PGC genes is largely restricted to cancer tissues, suggesting that diverse tumor types may harbor a general requirement for tightly regulating PGC expression.

**Table 1:** Validation of the PGC in Independent Data Sets.

Table 1A (Cancer)		Yu_Gastric&NPC (99^e^)		Wang_Breast (286)		Sotiriou_Breast (189)		Bild_Ovarian (125)		Bild_Lung (118)	
PGC	48^a^	28^b^/658^c^	**0** d	24/539	**0**	27/742	**5E-12**	21/488	**9E-11**	13/348	**3E-06**

		Phillips_Glioma (77)	Aronow_Colon (100)	NCI-60 (60)		Neve_BCL (51)	
PGC	48	20/585 **1E-17**	27/1125 **2E-8**	13/293	**5E-07**	9/410	**0.002**

Table 1B (Normal)		Symatlas (158) (79 Tissues)		Ge (36) (36 Tissues)	
PGC	48	6/345	0.07	10/542	0.01

a Number of genes in the original PGC signatures (e.g., PGC = 48 genes).

b Number of PGC signature genes that are tightly regulated in the test sets (CV<0.28).

c Total number of tightly regulated genes in the test sets (CV<0.28).

d Statistical significance of PGC signature enrichment, calculated by the hypergeometric distribution test. P-values with significance (<0.01) are highlighted in bold.

e Number of samples in the test data sets.

### PGC Genes Are Associated with Multiple Cancer Related Pathways

A pathway analysis revealed multiple highly significant interactions between the PGC genes and prevalent tumorigenic pathways. The top-scoring molecular network for the PGC comprised 11 PGC focus genes interacting either directly or indirectly with the well-known cancer-related transcription factors *Myc* and *TP53* (p = 10^−19^, see Methods) (Figure S1), and the most significantly enriched cellular functions in the PGC were cancer (p<0.0045), tumor morphology (p<0.0045) and cell cycle control (p<0.0045). The PGC was also significantly enriched in components related to integrin signaling (p = 2.33E^−04^; Figure S1), a complex signaling pathway implicated in both positive and negative regulation of tumor cell growth and cancer metastasis. Besides integrin signaling, other individual PGC genes, such as *RPS2* and *RPL7A*, have also been previously implicated in the control of cellular transformation, tumor growth, aggressiveness, and metastasis [35],[36]; while the PGC gene *MUS81* has recently been reported to interact with p53 to maintain genome stability [37]. Thus, an array of biological and functional evidences suggest that the PGC genes are likely to be involved in the activity of multiple cancer-related pathways, and not ubiquitous ‘housekeeping’ cellular functions. The full list of PGC genes is provided in Table S3.

### Subtle Alterations in PGC Expression Are Associated with Metastatic Capacity of Cancer Cells

**Figure 3:**
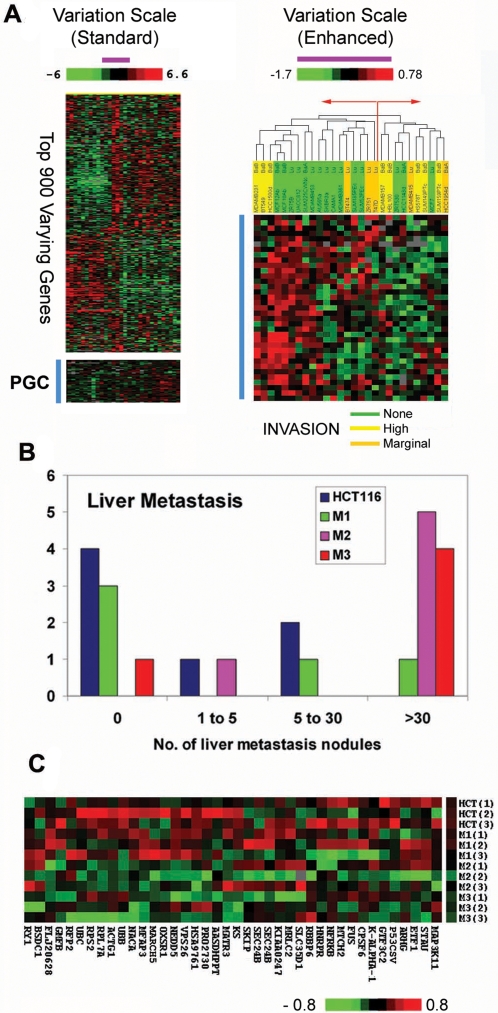
Subtle Alterations in PGC Expression are associated with invasion and metastasis *in vitro* and *in vivo*. A) Variation in PGC expression in breast cancer cell lines with differing invasive capacities. (Left) Expression heat-map depicting the range of PGC expression under a normal scale of variation, based on the top 900 varying genes in the data set (−6 to 6.6 fold, top scale bar). Under this normal scale, the PGC genes (blue column) are near black and show minimal expression variation across the cell lines. (Right) Expression heat-map depicting the range of PGC expression under a magnified scale of variation (−1.7 to 0.78 fold, compare purple bars between the left and right heat-maps). This heat-map represents the predominant pattern of gene expression, and does not contain 13 outlier PGC genes (see Figure S2). It worth noting that unsupervised hierarchical clustering based on the entire 48 PGC gene set was used to segregate the cell lines. Chi-square tests comparing the numbers of lines with no metastatic capacities in the two groups were assessed using the top level branch in the clustering tree (red arrows). Invasive capacities of the cell lines (none, high, marginal) were derived from Neve et al., 2006. B) Xenograft model of metastasis. Bar chart depicting the increasing metastatic potential of M1 to M3 cell lines compared to parental HCT116 cells. The x-axis depicts the number of metastatic modules observed per mouse, while the y-axis depicts the number of mice used in each experiment (n = 5 to 7 mice per cell line). C) Expression heat map showing expression of the PGC signature in HCT116, M1, M2, and M3 cell lines, aligned from top to bottom. Three independent biological replicates were profiled for each cell line. Red, black and green squares indicate high, moderate, and low expression respectively. Individual PGC gene names are listed below the heat-map. Note that the range of expression variation across the lines is very small, as shown by the scale-bar (−0.8 to 0.8 fold). This heat-map represents the predominant pattern of gene expression, and does not contain 8 outlier PGC genes (see Figure S2). Once again, the unsupervised hierarchical clustering based on the entire 48 PGC gene set was used to segregate the cell lines.

The tightness of PGC regulation in tumors might be explained if small alterations in the expression levels of these components are sufficient to cause significant phenotypic changes in tumors. We employed three experimental assays to address this possibility. First, we analyzed a set of colon cancer cell lines derived from either primary tumors or distant metastases from the same patient (SW480 and SW620), which have been shown to exhibit several phenotypic differences including metastatic potential [38],[39]. Using Gene Set Enrichment Analysis (GSEA, [40]), we found that PGC expression was subtly yet significantly decreased in highly metastatic SW620 cells compared to poorly metastatic SW480 cells (p<0.001, Table S4). Second, we then analyzed patterns of PGC expression in a cohort of 30 breast cancer cell lines, where the invasive capacity of each line had been previously measured by matrigel assays [32]. The PGC genes exhibited minimal expression variation across the lines when assessed using a standard range of expression variation, consistent with their being tightly regulated in cancers (Figure 3A, left heat-map). However, when the scale of variation was amplified, we identified by hierarchical clustering two groups of cell lines showing either subtly higher or lower levels of PGC expression (Figure 3A, right heat-map). Importantly, we again found that the majority of cell lines with high to moderate invasive abilities exhibited subtle yet significant decreased expression of the PGC genes compared to poorly invasive lines (p = 0.04, chi-square test, sample groups defined on the basis of the top-level branch point). To validate the robustness of this clustering by an alternative method, we then also performed independent k-means clustering (k = 2). Using k-means, 7 out of 8 highly invasive cell lines were clustered into one group together with 4 marginally or non-invasive cell lines (p = 0.01, chi-square test for high vs. marginal/non-invasive), consistent with the groupings observed by hierarchical clustering. Third, we conducted *in vivo* experiments using a murine xenograft model of metastasis, where poorly metastatic HCT116 colon cancer cells were injected into the spleens of nude mice, and metastatic liver tumor nodules were harvested 6 to 8 weeks later. The liver nodules were expanded in culture and re-passaged in mice to generate a panel of lines (M1, M2, and M3) with increasing levels of metastatic capacity (Figure 3B). Examining the gene expression profiles of these lines, we found that highly metastatic cells once again exhibited subtly decreased PGC expression compared to poorly metastatic HCT116 cells (p = 0.03, Figure 3B and Table S4). These results, based on three different experimental models of metastasis, collectively suggest that small alterations in PGC expression in tumors may be associated with potent differences in tumor physiology, specifically metastatic and invasive capacity.

### Functional Silencing of Multiple PGC Genes Enhance Cellular Invasion

**Figure 4:**
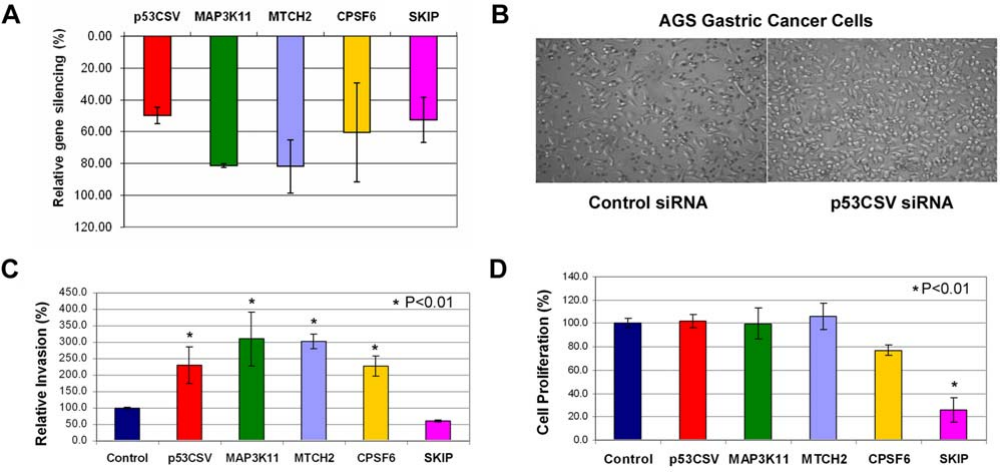
Reducing *PGC* gene expression by siRNA enhances the invasive behavior of AGS gastric cancer cells. A) Real-time PCR quantification of siRNA mediated knockdown efficiency of five PGC genes (*p53CSV*, *MAP3K11*, *MTCH2*, *CPSF6* and *SKIP*). The y-axis represents the percentage of relative silencing achieved by the different siRNA treatments. Relative silencing was calculated by comparing PGC gene expression levels between cells treated with either control or PGC target siRNAs. For each siRNA treatment, the expression levels of the PGC genes were normalized against the *GADPH* expression level. B) Representative photographs of AGS cells in the matrigel invasion assay. The left panel depicts control siRNA treated cells, while the right panel indicates *p53CSV* siRNA treated cells. Note the increased number of invading cells in the right panel. C) Summary graph of invasion effects caused by PGC gene silencing. Significant enhancements in cellular invasion were observed for *p53CSV*, *MAP3K11*, *MTCH2*, *CPSF6* (* symbols, P<0.01). P-values were calculated using a one-tailed t-test. D) Summary graph of cell proliferation effects caused by PGC gene silencing. Significant reductions in cell proliferation were only observed for the *SKIP* siRNA treatments. P-values were calculated using a one-tailed t-test.

To directly demonstrate the functional role of PGC genes in cellular invasion, we performed siRNA experiments where five PGC genes (*p53CSV*, *MAP3K11*, *MTCH2*, *CPSF6*, *SKIP*) were silenced in poorly-metastatic AGS gastric cancer cells. While *p53CSV* is a gene required for p53-mediated cell survival [41], its role in cancer is otherwise poorly understood. Furthermore, associations between *MAP3K11*, *MTCH2* and *CPSF6* to cancer have also not been previously reported. The siRNA treatments reduced the expression levels of these five PGC genes from 45%–80%, as assessed by quantitative real-time PCR (Figure 4A), and reductions in *p53CSV*, *MAP3K11*, *MTCH2* and *CPSF6* resulted in a significant enhancement of *in vitro* invasive activity as measured in a matrigel assay (p<0.01, one-tailed t-test, Figures 4B and 4C). Furthermore, *SKIP* siRNA treatment resulted in a significant inhibition of cellular proliferation in AGS cells (p<0.01, Figure 4D). It is worth noting that for at least two genes (*p53CSV* and *CPSF6*), a partial reduction of gene expression of 45–60% was able to trigger a significant change in invasive phenotype. To further demonstrate the generality of this phenomenon, we then knocked down *p53CSV* in another poorly-metastatic colon cancer cell line, HCT116 which we previously utilized in the xenograft assay. Again, the partial silencing of *p53CSV* expression significantly increased the invasion activity of HCT116 cells (Figure S3). These results suggest that the PGC genes may play roles in regulating cancer invasion and metastasis.

### Subtle Alterations in PGC Expression Are Associated with Clinical Outcome

**Figure 5:**
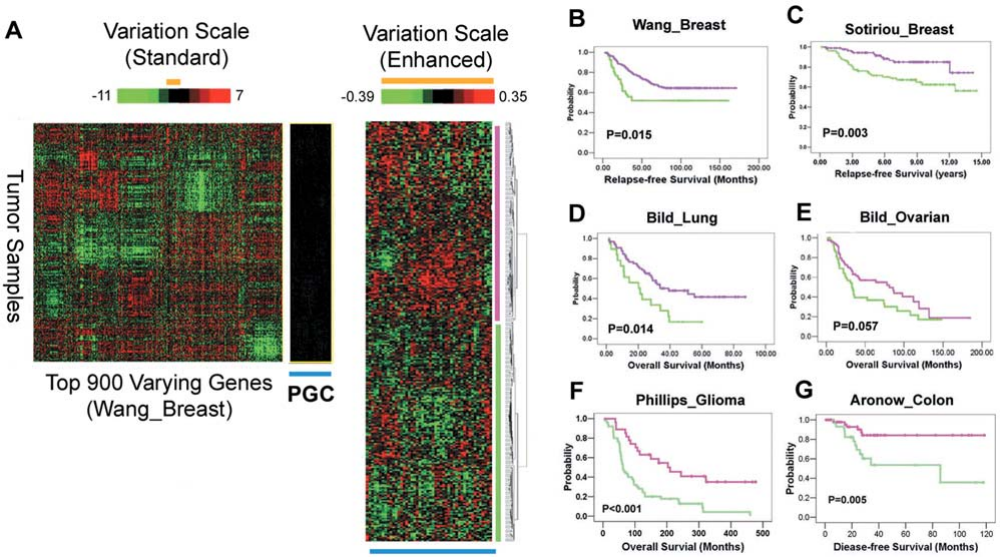
PGC Expression in Primary Tumors Predicts Clinical Outcome. A) Variation in PGC expression in primary breast tumors. (Left) Expression heat-map depicting the range of PGC expression under a normal scale of variation, as assessed by the top 900 varying genes in the Wang_Breast data set (−11 to 7 fold, top scale bar). Under this normal scale, the PGC genes (blue row) are near black and show minimal expression variation across the tumors. (Right) Expression heat-map depicting the range of PGC expression under a magnified scale of variation (−0.39 to 0.35 fold, compare orange bars between the left and right heat-maps). Unsupervised hierarchical clustering was used to segregate the tumors. All subsequent Kaplan-Meier analyses were performed between groups defined by the top level tree branch (purple and green samples). B)–E) Kaplan-Meier survival analysis of patient groups stratified by the PGC expression in primary tumors. Significantly distinct survival outcomes were observed in patients whose tumors express increased PGC levels compared to low-PGC expression patients, in the B) Wang_Breast set (p = 0.015), C) Sotiriou_Breast (p = 0.003), D) Bild_Lung (p = 0.014), and E) Bild_Ovarian cancer cohorts (p = 0.057), F) Phillips_Glioma cancer (p<0.001), and G) Aronow_Colon cancer cohorts (p = 0.005). The outcome metric was relapse-free survival for B), C), and G) and overall survival for D), E) and F).

To extend the potential role of precise PGC regulation to the clinical context, we asked if similar small changes in PGC expression might be associated with significant differences in patient survival and clinical outcome. We employed hierarchical clustering to group the tumors in each of the six data sets with survival data available by their overall level of PGC expression. A representative example is shown in Figure 5A. Once again, the PGC genes exhibited minimal expression variation across the tumors when assessed on a standard scale of expression variation, consistent with their being tightly regulated in tumors (Figure 5A, left heat-map). However, when the variation scale was amplified, we identified two groups of tumors showing either subtly higher or lower levels of PGC expression (Figure 5A, right heat-map). Remarkably, a Kaplan-Meier survival analysis revealed that in all six data sets, patients with tumors expressing PGC levels below the population average experienced significantly worse survival outcomes compared to patients with high-PGC expressing tumors (Figure 5B; all cases p<0.05 except in ovarian cancer set where p = 0.057, see Figure S4 for clustering groupings). We only observed comparable survival stratifications across the six data sets in 46 out of 10,000 randomly selected 48-member gene sets, arguing that the prognostic ability of the PGC is statistically unique. In a multivariate analysis, PGC expression behaved as an independent prognostic factor compared to other clinical variables in the breast and colon cancer cohorts, and was associated with tumor stage in ovarian, lung and glioma cancer patients (Table S5).

Importantly, the PGC exhibits very little overlap with other expression signatures reported to predict clinical behavior in multiple tumor types. A comparison of the PGC against a 128-gene metastasis signature (MS) [42], a 70-gene chromosomal instability signature (CIN70) [43], a cell cycle module [44], a wound response healing signature [45],[46], and multiple cell proliferation-related signatures (57–59) including a 874-gene cell cycle gene signature (CPS) [47], revealed that there was no direct overlap in gene content between the PGC and these other “multi-tumor” gene signatures, except for a one-gene overlap with the CIN70, and a four-gene overlap with the CPS, which was not statistically significant. This finding suggests that the specific gene content of the PGC is distinct from other previously described signatures. To ask if the PGC might target the same “poor prognosis” tumors as other published signatures capable of predicting clinical outcome in multiple tumor types, we then investigated the ability of the MS, CIN70, and CPS to stratify patient survival in the six data sets - none of these signatures exhibited comparable prognostic significance to the PGC across the six patient cohorts (data not shown). These observations suggest that the PGC is likely to target different molecular features and types of tumors than the aforementioned signatures.

### Conventional Microarray Analysis Methods Fail to Detect the Majority of the PGC

Previous gene expression studies comparing tumors and non-malignant tissues have typically employed microarray analysis algorithms such as t-tests with false positive correction or SAM [20]. Genes detected by such techniques typically require both differing mean expression levels and equivalent levels of variation between two cellular states (Figure S5). However, the PGC might not be detected by such conventional techniques, as PGC genes might not exhibit distinct mean expression levels between the two groups and only be associated with differing degrees of expression variation between tumors and controls (Figure S5). Indeed, performing SAM and t-tests on the training set only identified 27% of the original PGC, after multiple hypothesis correction, and the absolute mean expression levels of many PGC genes between tumors or non-malignant tissues were highly similar (Figure S5). To ask if the unequal distributions in expression variation might underlie the failure of the PGC genes to be identified by conventional techniques, we also analyzed the original training data set using Welch's test, an adaptation of Student's t-test intended for use with two groups having unequal variance. Again, 75% of the PGC genes failed to be detected as significant using Welch's test (data not shown). These findings suggest that conventional algorithms would likely have failed to detect the PGC, thereby providing a partial explanation as to why the PGC might have been missed in previous studies.

## Discussion

In this study, we identified a novel cassette of genes exhibiting tumor-specific precise regulation in multiple cancer tissues. Our ability to discern the PGC was facilitated by the use of an analysis method focused on expression variance rather than expression levels. The reduced variance of the PGC in tumors is unlikely to be a technical artifact of the Affymetrix platform, as it was not related to probe selection, data normalization, absolute high or low expression levels in either tumors or non-malignant tissues, or sample set. Using both rigorous cross-validation (RSS) and multiple independent validations, we found the PGC to be robust to alterations in training set composition and repeatedly observed in diverse malignant tumor types, including several tissue types not present in the original training data. Importantly, the PGC failed to demonstrate tight regulation in several non-malignant tissue data sets, arguing that its control is cancer-specific. Interestingly, even though it was not a specific requirement in our initial analysis, the majority of PGC genes exhibited similar mean expression levels in both tumors and non-malignant tissues. This absence of a distinct difference in mean expression values resulted in the failure of standard microarray analysis methods (e.g., t-test) to detect the majority of PGC genes when applied to the same training data set. Furthermore, a standard practice in microarray data processing is to filter out genes exhibiting low variation prior to clustering or statistical analysis - such filtering would inevitably lead to a bias towards differentially expressed genes and prevent the discovery of the PGC.

One potential concern might be that the PGC genes simply reflect the activity of tissue-specific gene expression. However, five findings argue against this possibility. First, while dedifferentiated cancer cells frequently exhibit a loss of tissue-specific gene expression (Rhodes et al. 2004); such a loss would typically result in tissue-specific genes being down-regulated in their absolute expression levels compared to normal tissues. In contrast, the PGC genes do not exhibit significant differences in their absolute expression levels between cancers and normal tissues (Figure 2B). Second, the reduced variation of the PGC genes was consistently observed in multiple independent sets from diverse tissues (e.g. gliomas, lung, breast), including a data set (NCC) that combined tissues from two different sources (gastric and NPC tumors). Third, the PGC genes also showed reduced expression variation in the NCI60 test set - a mixture of cancer cell lines from 9 different tissue types. Fourth, the PGC genes consistently exhibited reduced expression variation in the repeated random sampling (RSS) cross-validation assay, where we tested 1000 distinct training set and independent test sets composed of mixed tissue types (Figure 2D). Fifth, even within each of the six tissue types in the training set (liver, colon, esophagus, thyroid, lung, and breast), the majority of the PGC genes (70%) are not differentially expressed within tumors and normals (p>0.01, t-test) (YK, data not shown). Taken collectively, it is unlikely that the consistency of the PGC would have been observed if its reduced expression variation was solely due to tissue-specific expression, supporting the notion that the PGC genes are likely to be distinct from the conventional differentially expressed gene signatures described in most microarray studies.

One possible explanation for why certain genes may require precise control is if they regulate or are involved in balancing disparate downstream pathways possessing mutually opposing activities. In cancers, the successful establishment of a malignant tumor involves multiple pro- and anti-oncogenic forces involved in cell proliferation, apoptosis, cell death, senescence, cell adhesion, and motility, all of which require delicate balance by different genetic components. For example, while loss of Ras signaling is lethal, aberrant signaling through this pathway is important for cancer development but can also drive cells into either senescence or cell proliferation, depending on cellular context [48],[49]. Another good example is the anti-apoptotic gene Akt/PKB (protein kinase B), which when constitutively activated reduced metastases in mice and inhibited the invasion of breast cancer cells [50],[51], indicating its involvement in multiple cancer pathways. Reassuringly, similar examples of balanced coordinator genes are also seen in the cohort of PGC genes. The PGC gene *FUS1* (also known as *FUS*) has been reported as a tumor suppressor gene in lung and breast cancer [52] and a pro-oncogene in leukemia [53]. Oxidative stress, which may play an important role in cancer progression and the regulation of cancer metastasis [54], is dependent upon the critical balance between intracellular hydrogen peroxide H_2_O_2_ and superoxide O_2_^−^. Two PGC genes - *p53CSV* and *KIAA0247* have been reported to be induced in response to oxidative stress [55], and may influence this balance and the response of tumor cells to apoptotic stimuli [56]. It is also worth noting that the PGC was significantly overrepresented in components of the integrin signaling pathway – a highly complex process involving multiple related family members with roles in many cellular functions, including ERK/MAPK and JNK/SAPK regulated gene expression, cell motility, cytoskeletal interactions, and PI3K and Wnt pathway signaling [57]. In metastasis, integrins are crucial for cell invasion and migration, not only for physically tethering cells to the matrix, but also for sending and receiving molecular signals regulating these processes [57]. Moreover, while some groups have proposed that increased integrin expression could promote malignant behavior by enhancing tissue stiffness [58], other groups have suggested that loss of integrins may promote tumor invasion and metastasis [59]. The complexity of integrin family members and their pathway components also provides a plausible explanation for why even subtle alterations in PGC expression are associated with distinct and measurable changes in metastatic behaviour in both experimental models of metastasis and clinical outcome.

What might be the mechanistic basis of precise PGC regulation? At a general level, many precisely-regulated genes are likely to possess complex regulatory systems for tightly controlling expression levels, to rapidly sense and adapt to dynamic perturbations in both the internal and external environment [60]. Such mechanisms could involve the use of both positive and negative feedback loops, analogous to the circuitry utilized by the LacI/O bacterial system to ensure precise expression [61], but in cancers could also involve eukaryote-specific mechanisms like epigenetic modifications (DNA methylation or chromatin modifications), microRNA regulation, or transcription factor binding. Interestingly, in a preliminary analysis, we attempted to extend our observations from the pathway analysis showing an association of several PGC genes with both *Myc* and *TP53*. Specifically, we investigated whole-genome transcription factor binding data for *Myc* and *TP53* [62], and found that the PGC genes were weakly but significantly associated with *Myc* binding sites under *Myc*-overexpressed (tumorigenic) conditions (p = 0.04) but not under physiological conditions (p = 0.3) (Table S6). These preliminary results raise the possibility that transcription factor binding, specifically Myc binding, may constitute one possible mechanism for PGC regulation in cancer cells. However, deciphering the mechanism of PGC regulation will undoubtedly require further research.

Cancers have been proposed to possess robustness mechanisms for protection against various therapeutic perturbations and naturally occurring microenviromental (e.g., hypoxia) and immune responses. However, many complex systems have evolved to exhibit a ‘robust yet fragile’ structure [63],[64], and it has been proposed that studying mechanisms of cancer-specific robustness and accompanying fragilities might prove useful for the development of novel targeted therapies [65]–[67]. The PGC gene cassette reported here may indicate such fragilities in the network of tumor cells, as subtle alterations on these components significantly affected the cellular behavior of cancer cells. Beyond cancer, this approach is conceptually applicable and easily transportable to other disease conditions where gene expression data is available. It will be interesting to explore if the approach will also prove informative in identifying genes and pathways with important roles in other human pathophysiologies.

## Materials and Methods

### Microarray Data Sets

The training data set contained 270 primary human tumors (Lung = 18, Thyroid = 35, Liver = 9, Esophagus = 16, Colon = 9, Breast = 183) and 71 adjacent non-malignant tissues (Lung = 12, Thyroid = 16, Liver = 8, Esophagus = 13, Colon = 9, Breast = 13) obtained from the Tissue Repository of the National Cancer Centre of Singapore (NCCS). The phrase ‘non-malignant’ instead of ‘normal’ was used to describe the control tissues in the training set, as they were also obtained from cancer patients. Institutional approvals were obtained from the NCCS Tissue Repository and Ethics Committees. Descriptions of sample collection protocols, archiving, and histological assessments are presented in the Text S1. RNA was extracted from the tissues using Trizol reagent (Invitrogen, Carlsbad, CA) and processed for microarray hybridizations on Affymetrix U133A Genechips according to the manufacturer's instructions (Affymetrix Inc., Santa Clara, CA). The expression data has been deposited into the Gene Expression Omnibus (GEO) database (GSE5364).

### Data Preprocessing

Raw Genechip scans were processed using either the MAS5 algorithm (Affymetrix) normalized by median-centering (GeneData, Basel, Switzerland), or by robust multiple chip analysis (RMA) [24],[25] (see Results). To identify reliably measured genes, we discarded probes with <80% present values (P-call <80%) across the training set samples. For genes with multiple probes, we selected the best-match probes (to targets) represented by a “_at” extension. For genes with multiple “_at” extension probes, the probe with the highest P-call rate (i.e., the highest valid value proportion) was used. The final pre-processed training set comprises 5729 unique genes, each represented by a single probe.

### Coefficient of Variance (CV)

Gene expression CVs (standard deviation divided by the mean expression level) were used to compute the variability of expression for each gene. Based on the global distribution of CVs in the training set, we selected an empirical threshold of CV_T_ = 0.28 below which a gene was considered to be tightly regulated (see Results). Prior to comparing gene CVs between populations, we also confirmed that the global CV distributions for different sample cohorts (i.e., tumor or non-malignant) were similar.

### Repeated Random Sampling (RRS)

To estimate the probability that the PGC signatures might be generated by chance, we randomly shuffled the class labels (i.e., tumor or non-malignant) of the training set to generate multiple class-permuted sample sets and determined the frequency a particular PGC gene could be re-identified in situations where the sample labels were shuffled. Repeated Random Sampling (RRS), a rigorous cross-validation strategy described in [27], was also used to determine the influence of particular training set compositions on selecting specific signature genes. Detailed descriptions of the class permutation and RSS tests are provided in the Text S1.

### Validation of the PGC in Test Sets

The hypergeometric distribution was used to test if the PGC genes were significantly over represented in the population of tightly controlled genes in each test set. First, we identified genes exhibiting tightly controlled expression in the test set, using the CV_T_ threshold cut-off (CV(Test)<CV_T_). Second, we determined the overlap between the PGC gene signatures and the population of tightly regulated genes in the test set, and the hypergeometric distribution test was used to calculate the significance of the overlap. Significance was defined as p<0.01.

### Pathway Analysis

We used Ingenuity Pathway Analysis (IPA, Ingenuity Systems) to identify molecular networks, cellular functions, and signaling pathways associated with the PGC. The various networks were displayed as nodes (genes) and edges (biological relationships between genes). We also used IPA to identify cellular functions and signaling pathways that were significantly enriched in the PGC. The significance of a pathway association is reflected by a Fisher's exact test p-value, indicating the likelihood that the pathway would have been identified by random chance.

### Invasion and Proliferation Assays

AGS gastric cancer cells and HCT116 colon cancer cells were cultured according to American Type Culture Collection (ATCC) recommendations. Cells were transfected with either siRNA pools of representative PGC genes p53*CSV*, *MAP3K11*, *MTCH2*, *CPSF6* and *SKIP* (Dharmacon, Lafayette, CO) or non-targeting siRNA controls at 100 nM concentration, using oligofectamine reagent (Invitrogen) at 0 and 24 hr time points, in 6 well culture plates. Gene silencing was verified by Real time PCR. Invasion assays were performed using Biocoat matrigel invasion chambers (BD Biosciences, Bedford, MA) as recommended by the manufacturer. 48 hrs after siRNA transfection, equal numbers of target gene siRNA transfected cells and non-targeting siRNA transfected cells were placed in the invasion chambers, and after 24 hrs cells that successfully invaded through the matrigel invasion chambers were scored. Each experiment was repeated thrice and the percentage of invasion was calculated by comparing against the non-targeting siRNA transfected cells. To assay cell proliferation, AGS cells transfected with the PGC genes and non-targeting control siRNA in 6 well culture plates were harvested at 96 hrs after siRNA transfection and counted. Experiments were performed thrice.

### Quantitative Real-time PCR

Total RNA was reverse transcribed using Taqman Reverse Transcription Reagent kit (Applied Biosystems, Foster City, CA) and quantitative PCR was performed using the following Taqman probes: *p53CSV* (Hs00429934_g1); *MAP3K11* (Hs00176759_m1); *MTCH2* (Hs00819318_g1); *CPSF6* (Hs00199668_m1); *SKIP* (Hs00273351_m1), on a 7900HT Fast Real time system (Applied Biosystems, Foster City, CA). Taqman GAPDH probes (glyceraldehyde phosphate dehydrogenase) (Hs99999905_m1) were used as internal controls. All samples were run in triplicates.

### Experimental Systems of Cancer Metastasis and Invasion

(A) Colorectal cancer model : this comprises two colon cancer cell lines derived from either primary or distant metastases from the same patient (SW480 and SW620). SW480 and SW620 cells exhibit several phenotypic differences including metastatic potential [38],[39]. Gene Set Enrichment Analysis (GSEA) was performed as described in [40]. (B) Breast cancer panel: this comprises a panel of 51 breast cancer cell lines for which gene expression data is available [32], and where the relative invasive capability of 30 lines has been measured using matrigel assays [32]. (C) Murine assay: this comprises an *in vivo* passage model where poorly metastatic HCT116 colon cancer cells were injected into mouse spleens, and subsequent hepatic metastases were harvested to generate increasingly metastatic cellular variants. Details of this model are provided in the Text S1. The animal work performed was approved by the National University of Singapore Institutional Animal Care and Use Committee (NUS IACUC). Cells obtained from the hepatic metastatic nodules after the first passage were named M1, and the selection procedure was repeated twice to obtain the M2 and M3 cell lines. Three independent replicates were profiled for each cell line.

### Clinical Outcome in Primary Patient Cohorts

Hierarchical clustering (average linkage metric with Pearson correlation) was used to cluster tumors into different groups on the basis of their PGC expression levels. Kaplan-Meier analysis (SPPC, Chicago) was used for survival comparisons between the tumor groups. P-values were calculated using the Log-rank test.

## Supporting Information

Figure S1 Pathway analysis of PGC genes.
(1.30 MB DOC)

Figure S2 Clustering of cell lines based on the 48 gene set.
(0.16 MB DOC)

Figure S3 Reducing p53CSV expression by siRNA enhances the invasive behavior of HCT116 colon cancer cells.
(0.38 MB DOC)

Figure S4 Heatmaps of clustering of PGC in five tumor data sets.
(1.50 MB DOC)

Figure S5 Failure of PGC detection by conventional microarray analysis algorithms.
(0.19 MB DOC)

Table S1 Cross-validation performance of the PGC gene set under a range of CV threshold values.
(0.03 MB DOC)

Table S2 Summary of independent test data sets.
(0.03 MB DOC)

Table S3 The PGC gene list.
(0.07 MB DOC)

Table S4 Association of the PGC expression with metastatic activity.
(0.03 MB DOC)

Table S5 Multivariate analysis for the PGC in primary tumors.
(0.06 MB DOC)

Table S6 Association between PGC genes and Myc/p53 genome binding loci.
(0.03 MB DOC)

Text S1 Supplementary methods.
(0.09 MB DOC)
