# Supplementary material for: A Precisely Regulated Gene Expression Cassette Potently Modulates Metastasis and Survival in Multiple Solid Cancers
Source: PLoS Genet. 2008 Jul 18;4(7):e1000129. doi: 10.1371/journal.pgen.1000129 (PMC2444049; doi:10.1371/journal.pgen.1000129)

**Figure S1. Pathway analysis of PGC Genes**

**A)** Top scoring PGC-associated networks using Ingenuity Pathway Analysis. PGC genes are marked in bold with gray colors. The networks are graphically displayed as nodes (genes/gene products) and edges (biological relationships between nodes). As described in the Legend (top left panel), Nodes are displayed using various shapes that represent the functional class of the gene product (eg, circle for transcriptional factor, diamond for enzyme, triangle for phosphatase and reversing triangle for Kinase). Edges are displayed with various labels that describe the nature of the relationship between the nodes (e.g., B for binding, E for expression and PP for protein-protein binding). The length of an edge reflects the evidence supporting that node-to-node relationship, in that edges supported by more articles from the literature are shorter. Higher-level functions for this network are reported (see Methods for details).

**B**) and **C**) PGC genes in the integrin pathway: **B**) Overview of the integrin signaling pathway; **C**) Zoom-in on the cell motility branch of the pathway, which contains PGC genes. The PGC genes are highlighted in grey.


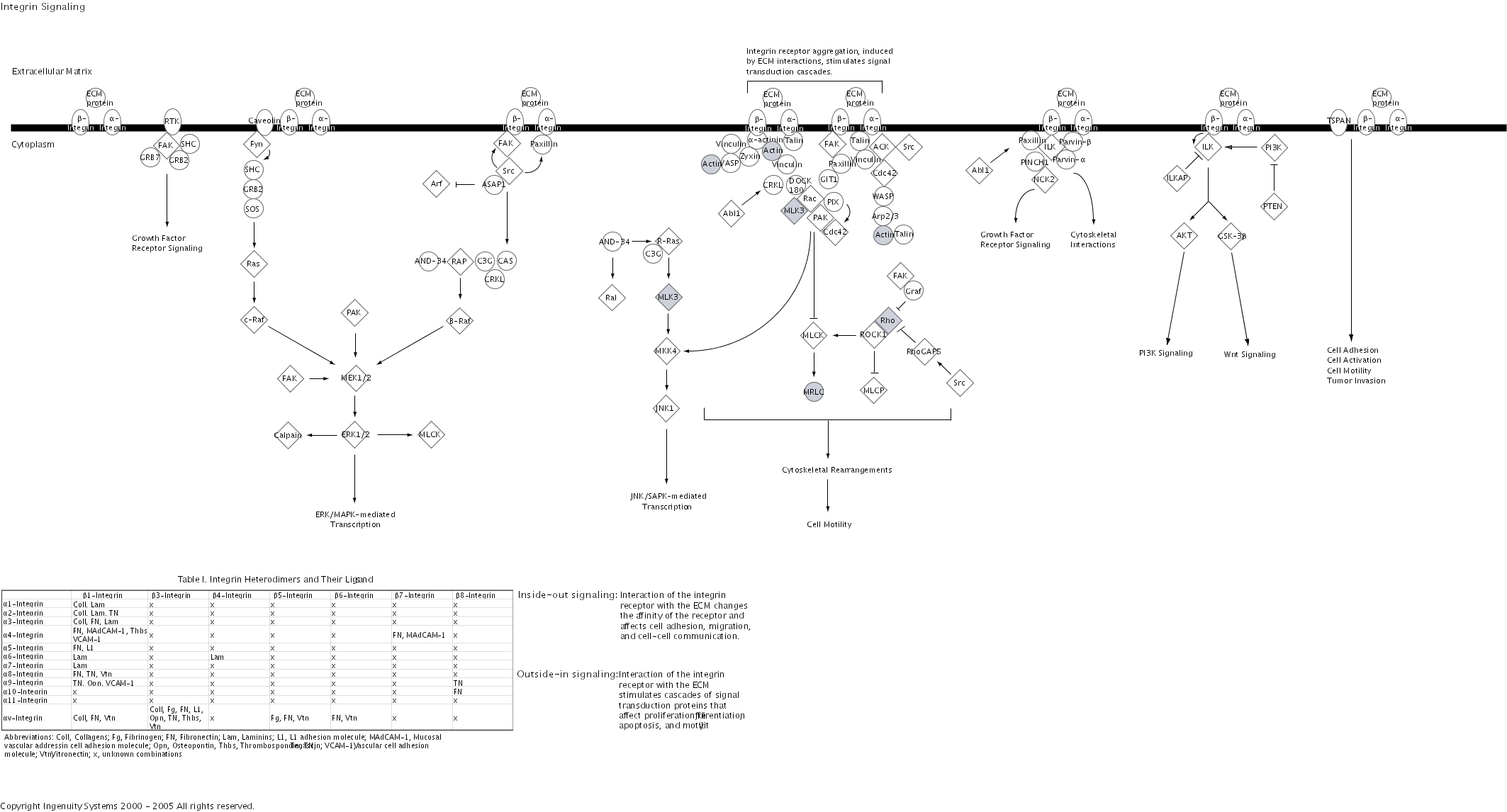


**A)**


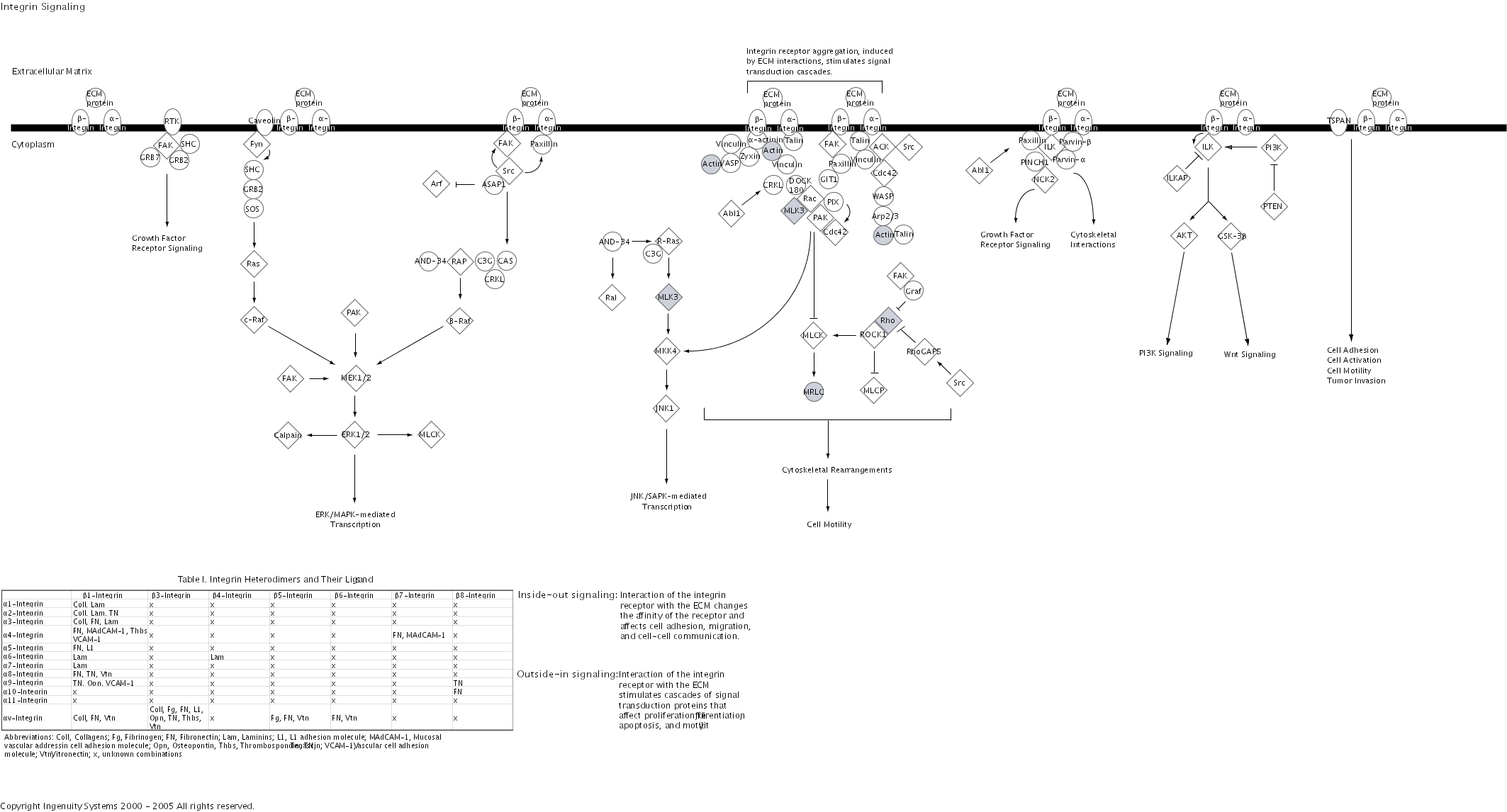

Supplement: Figure S1 — Pathway analysis of PGC genes. (1.30 MB DOC) [file pgen.1000129.s001.doc]
