# Supplementary material for: A Precisely Regulated Gene Expression Cassette Potently Modulates Metastasis and Survival in Multiple Solid Cancers
Source: PLoS Genet. 2008 Jul 18;4(7):e1000129. doi: 10.1371/journal.pgen.1000129 (PMC2444049; doi:10.1371/journal.pgen.1000129)

**Figure S2 : Clustering of Cell Lines Based on the 48 Gene Set.**

While the clustering of the samples for Figures 3A and 3B was performed using the entire 48-member gene set, a few ‘outlier’ genes (13 genes for 3A and 8 genes for 3B) were removed from the figure since our attention was to focus on the dominant focused on the dominant PGC expression pattern. Here, we have now included the heatmaps with the complete set of 48 PGC genes. It is important to note that the clustering of cell lines is based on the expression pattern of the entire set of 48 PGC genes.

3A : Breast Cell Lines 3B : Mouse Xenograft


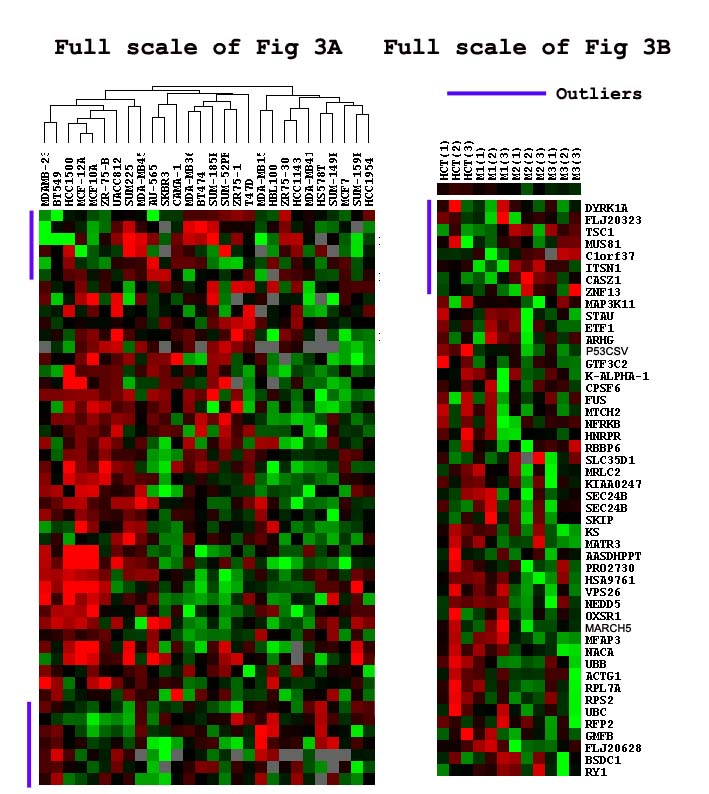

Supplement: Figure S2 — Clustering of cell lines based on the 48 gene set. (0.16 MB DOC) [file pgen.1000129.s002.doc]
