# Supplementary material for: A Precisely Regulated Gene Expression Cassette Potently Modulates Metastasis and Survival in Multiple Solid Cancers
Source: PLoS Genet. 2008 Jul 18;4(7):e1000129. doi: 10.1371/journal.pgen.1000129 (PMC2444049; doi:10.1371/journal.pgen.1000129)
Supplement: Figure S3 — Reducing p53CSV expression by siRNA enhances the invasive behavior of HCT116 colon cancer cells. (0.38 MB DOC) [file pgen.1000129.s003.doc]

**Figure S3. Reducing p53CSV expression by siRNA enhances the invasive behavior of HCT116 colon cancer cells**

A) siRNA mediated knockdown of *p53CSV* in HCT116 cells. RT-PCR figure showing silencing of *p53CSV* gene in HCT116 cells. GAPDH was used as the internal control.

B) Representative photographs of HCT116 cells in the matrigel invasion assay. The left panel depicts control siRNA treated cells, while the right panel indicates *p53CSV* siRNA treated cells.

C) p53CSV silencing increases the invasive behavior of HCT116 cells in matrigel invasion assays. Relative invasion was scored with reference to control siRNA transfected cells. The average and range derived from three independent experiments are indicated. The significance of the association is p=0.003 (one-tailed t-test).
