# Supplementary material for: A Precisely Regulated Gene Expression Cassette Potently Modulates Metastasis and Survival in Multiple Solid Cancers
Source: PLoS Genet. 2008 Jul 18;4(7):e1000129. doi: 10.1371/journal.pgen.1000129 (PMC2444049; doi:10.1371/journal.pgen.1000129)

**Figure S4. Heatmaps of clustering of PGC in five tumor data sets**

Expression heatmaps of clustering of PGC on five tumor data sets. The survival analysis, which are shown in Figure 5 (main text), were performed based on the results of these clustering. The two groups of tumors were defined based on the top level node in the dendrogram. The clustering of Sotirioi_Breast data set was performed in four separate subsets (see Supplementary Methods for details). The color bar (purple and gree line) is corresponding to the survive curves in Figure 5.


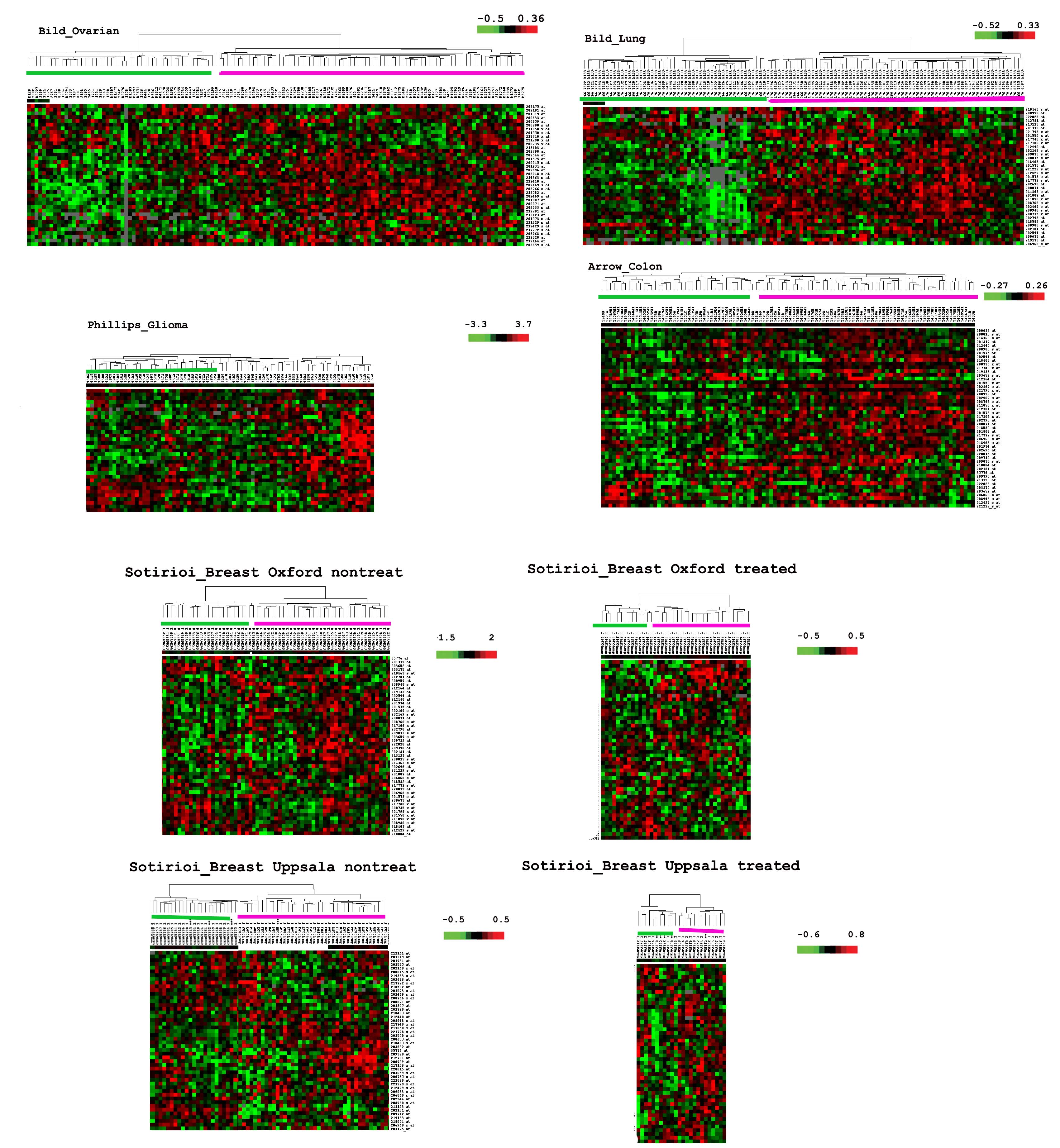

Supplement: Figure S4 — Heatmaps of clustering of PGC in five tumor data sets. (1.50 MB DOC) [file pgen.1000129.s004.doc]
