# Supplementary material for: A Precisely Regulated Gene Expression Cassette Potently Modulates Metastasis and Survival in Multiple Solid Cancers
Source: PLoS Genet. 2008 Jul 18;4(7):e1000129. doi: 10.1371/journal.pgen.1000129 (PMC2444049; doi:10.1371/journal.pgen.1000129)
Supplement: Figure S5 — Failure of PGC detection by conventional microarray analysis algorithms. (0.19 MB DOC) [file pgen.1000129.s005.doc]

**Figure S5. Failure of PGC Detection by Conventional Microarray Analysis Algorithms**

**A**) Example of a gene with differential absolute expression in distinct conditions. The mean expression level is greater in phenotype 1 (blue) compared to phenotype 2 (red), but the mean expression variation (CV) being the same in both conditions. The Y-axis depicts an arbitrary range of expression values.

**B**) Example of a gene with differential gene expression variation in different phenotypes. The expression variation is lower in phenotype 1 compared to phenotype 2, despite the ean expression level being similar in both conditions. The frequency histogram of expression values (right panel) demonstrates that the distribution of expression values in phenotype 2 is narrower than in phenotype 1.

**C**) Student’s t-test on the PGC: PGC were grouped into two categories based on their significance of t-test (p<0.05 for red spots; and p>0.05 for blue spots), after correction for multiple hypotheses using the Benjamini-Hochberg algorithm. The PGC genes were plotted based on the expression level in tumors (X-axis) and in controls (Y-axis).

**Figure S2**


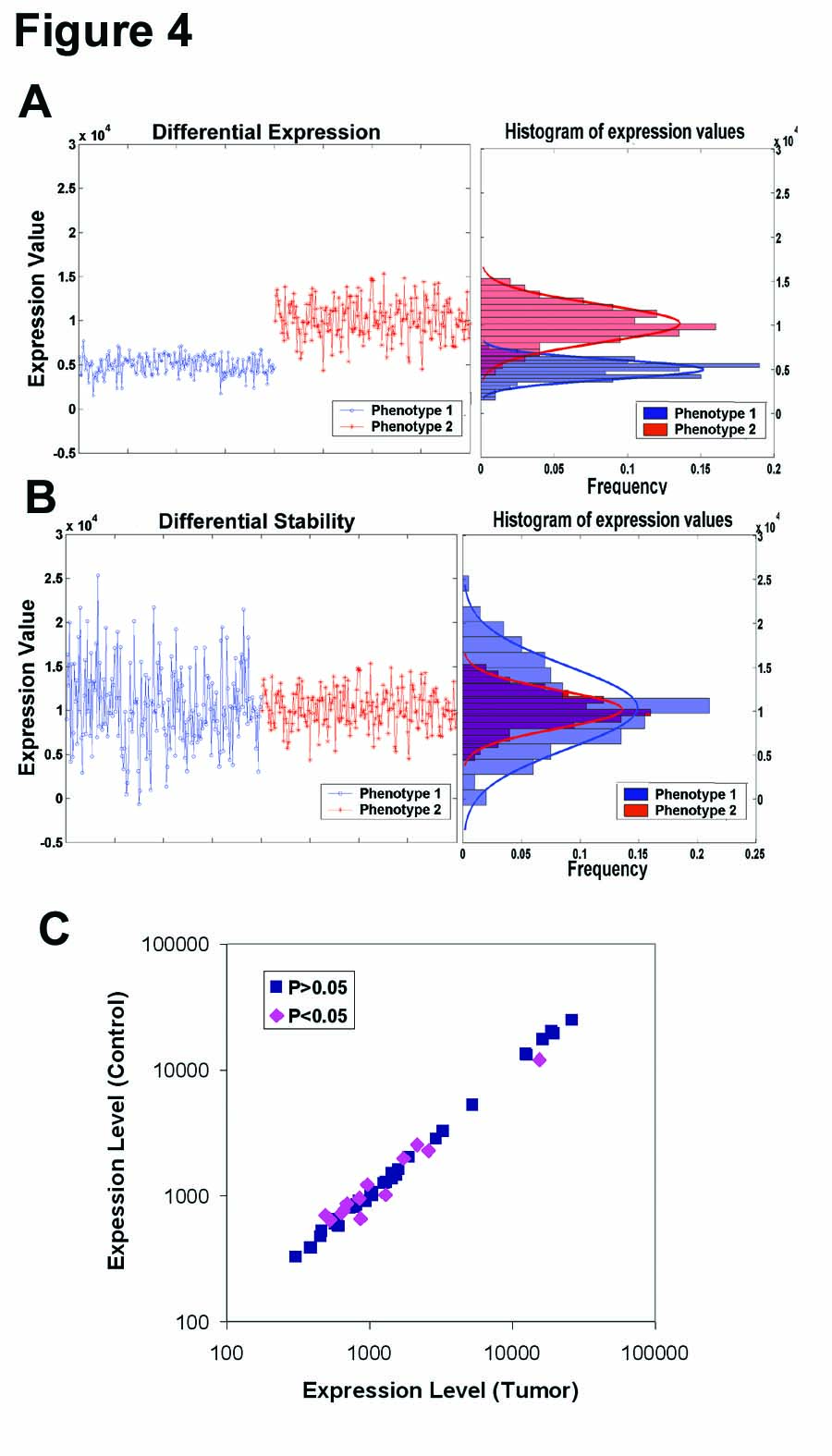


**Figure S5**
