# Supplementary material for: A Precisely Regulated Gene Expression Cassette Potently Modulates Metastasis and Survival in Multiple Solid Cancers
Source: PLoS Genet. 2008 Jul 18;4(7):e1000129. doi: 10.1371/journal.pgen.1000129 (PMC2444049; doi:10.1371/journal.pgen.1000129)
Supplement: Table S1 — Cross-validation performance of the PGC gene set under a range of CV threshold values. (0.03 MB DOC) [file pgen.1000129.s006.doc]

**Table S1.** **Cross-validation performance of the PGC gene set under a range of CV_T_ threshold values**

| **CV_T_** | **PGC** | **PGC in Tumor** | **PGC in Control** |
| --- | --- | --- | --- |
| 0.24 | 10 | 25% | 93% |
| 0.25 | 17 | 42% | 98% |
| 0.26 | 16 | 60% | 99% |
| 0.27 | 29 | 77% | 100% |
| 0.28 | 48 | 80% | 100% |
| 0.29 | 57 | 82% | 100% |
| 0.3 | 64 | 88% | 100% |

Note: The cross-validation procedures were the same as the repeated random sampling (RSS) test used in the Main text (see Methods). For each cut-off, the cross-validation was run 100 times.

The percentages indicate: 1) true positive rate (1^st^ column): how many times PGC is significantly enriched (p<0.01) in genes tightly regulated in the corresponding test set (PGC->Tumor); and 2) true negative rate (2^nd^ column): how many times PGC is NOT significantly enriched (p<0.01) in genes tightly regulated in the unmatched test set (PGC->Control). Within the CV_T_ range (0.26~0.3), we obtained similar levels of sensitivity and specificity in the PGC signatures.
