# Supplementary material for: A Precisely Regulated Gene Expression Cassette Potently Modulates Metastasis and Survival in Multiple Solid Cancers
Source: PLoS Genet. 2008 Jul 18;4(7):e1000129. doi: 10.1371/journal.pgen.1000129 (PMC2444049; doi:10.1371/journal.pgen.1000129)
Supplement: Table S2 — Summary of independent test data sets. (0.03 MB DOC) [file pgen.1000129.s007.doc]

**Table S2. Summary of Independent Test Data Sets**

These include I) an in-house set of 53 gastric tumors and 46 nasopharyngeal carcinomas (Yu_Gastric&NPC); II) a cohort of 286 lymph-node-negative breast cancer patients (Wang_Breast data set; GEO accession number: GSE2034); III) 189 invasive breast carcinomas from two research institutions (Sotiriou_Breast data set; GSE2990); IV) 125 ovarian tumors (Bild_Ovarian; GSE3149); V) 118 lung tumors (Bild_Lung; GSE3141); VI) 77 brain tumors (Phillips_Glimo; GSE4271); VII) 100 colon tumors (Aronow_Colon; GSE5206); VIII) a collection of 60 cancer cell lines (NCI60, http://symatlas.gnf.org/SymAtlas/); and VIIII) a separate collection of 51 breast cancer cell lines (Neve_BCL). Detailed descriptions of each data set are provided in the corresponding references.

| **Data Set** | **Yu_Gastric&NPC** | **Wang_Breast** | **Sotiriou_Breast** | **Bild_Ovarian** | **Bild_Lung** |
| --- | --- | --- | --- | --- | --- |
| **#Samples** | 99 (53 Gastric + 46 NPC) | 286 | 189 | 125 | 118 |
| **Source** | In-house | GSE2034 | GSE2990 | GSE3149 | GSE3141 |
| **Data Set** | **Phillips_Glioma** | **Aronow_Colon** | **NCI-60** | **Neve_BCL** | **Total (Cancer)** |
| **#Samples** | 77 | 100 | 60 | 51 | **1105** |
| **Source** | GSE4271 | GSE5206 | SymAtlas | Supplementary Data |  |
| **Data Set** | **Symatlas** | **Ge** | **Total**  **(Normal)** |  |  |
| **#Samples** | 158 | 36 | **194** |  |  |
| **Source** | SymAtlas | Supplementary Data |  |  |  |
