# Supplementary material for: A Precisely Regulated Gene Expression Cassette Potently Modulates Metastasis and Survival in Multiple Solid Cancers
Source: PLoS Genet. 2008 Jul 18;4(7):e1000129. doi: 10.1371/journal.pgen.1000129 (PMC2444049; doi:10.1371/journal.pgen.1000129)
Supplement: Table S3 — The PGC gene list. (0.07 MB DOC) [file pgen.1000129.s008.doc]

Table S3. The PGC Gene List. Gene Symbols were downloaded from the Affymetrix website.

| **Probe Set ID** | **Gene Symbol** | **Repeatedly Selected in Table 1^a^** | **Correlation with Metastasis Models ^b^** |
| --- | --- | --- | --- |
| 200633_at | UBB | X |  |
| 200015_s_at | SEPT2 | X |  |
| 200071_at | SMNDC1 |  |  |
| 200735_x_at | NACA | X |  |
| 200959_at | FUS |  |  |
| 201319_at | MRCL3 |  |  |
| 201550_x_at | ACTG1 | X |  |
| 201573_s_at | ETF1 |  | X |
| 201575_at | SKIIP |  |  |
| 201807_at | VPS26 |  |  |
| 201934_at | PRO2730 |  |  |
| 202169_s_at | AASDHPPT | |  |
| 202181_at | KIAA0247 |  | X |
| 202469_s_at | CPSF6 | X |  |
| 202544_at | GMFB |  |  |
| 202696_at | OXSR1 | X |  |
| 202798_at | SEC24B |  | X |
| 203175_at | RHOG |  |  |
| 203652_at | MAP3K11 |  |  |
| 203659_s_at | RFP2 | X |  |
| 206860_s_at | FLJ20323 |  | X |
| 206968_s_at | NFRKB |  |  |
| 208766_s_at | HNRPR | X |  |
| 208948_s_at | STAU |  | X |
| 208980_s_at | UBC | X |  |
| 209033_s_at | DYRK1A |  | X |
| 209390_at | TSC1 |  |  |
| 209712_at | SLC35D1 |  |  |
| 211058_x_at | K-ALPHA-1 | X |  |
| 212164_at | C1orf37 |  |  |
| 212429_s_at | GTF3C2 | X |  |
| 212440_at | RY1 |  |  |
| 212781_at | RBBP6 |  |  |
| 213123_at | MFAP3 |  | X |
| 214363_s_at | MATR3 | X |  |
| 217106_x_at | HSA9761 |  |  |
| 217740_x_at | RPL7A |  |  |
| 217772_s_at | MTCH2 |  |  |
| 218004_at | FLJ10276 | X | X |
| 218403_at | P53CSV | X |  |
| 218463_s_at | MUS81 | X |  |
| 218582_at | MARCH3 |  | X |
| 219133_at | KS | X | X |
| 220015_at | FLJ20321 | X |  |
| 221229_s_at | FLJ20628 | X | X |
| 221798_x_at | RPS2 | X |  |
| 222028_at | ZNF45 |  |  |
| 35776_at | ITSN1 |  |  |

**^a^** PGC genes showing reduced expression variation in more than half of the nine cancer test sets (Table 1, main text).

**^b^** PGC genes whose expression levels were correlated with metastasis in all three experimental metastasis models
