# Supplementary material for: A Precisely Regulated Gene Expression Cassette Potently Modulates Metastasis and Survival in Multiple Solid Cancers
Source: PLoS Genet. 2008 Jul 18;4(7):e1000129. doi: 10.1371/journal.pgen.1000129 (PMC2444049; doi:10.1371/journal.pgen.1000129)
Supplement: Table S4 — Association of the PGC expression with metastatic activity. (0.03 MB DOC) [file pgen.1000129.s009.doc]

**Table S4 Association of the PGC expression with Metastatic Activity**

|  | **Xenograft**^a^ |  | **Human**^b^ |
| --- | --- | --- | --- |
|  | p-value |  | p-value |
| PGC | **0.03** |  | **<0.001** |

In both cases, PGC expression is inversely correlated with metastatic activity.

^a^Associations between PGC expression and metastatic vector were calculated using Gene Set Enrichment Analysis (GSEA) (Supplementary Methods).
^b^Comparison of PGC expression to cellular phenotype in isogenic colorectal cancer (CRC) cell lines from the same patient obtained from either primary or metastasis sites.

P-values with significance (p<0.05) are highlighted in bold.

**b) Detailed results of GSEA on each of the nine metastasis vectors [1 2-4 7-9 10]**.

|  | **Summary of nine metastasis vectors** | |  |
| --- | --- | --- | --- |
|  | p-value (median) | p-value  (range) |  |
| PGC | **0.045** | 0.008 - 0.15 |  |

Note: The association between expression level of PGC and metastasis activity was calculated by using GSEA. P-values with significance (<0.05) are highlighted in bold.
