# Supplementary material for: A Precisely Regulated Gene Expression Cassette Potently Modulates Metastasis and Survival in Multiple Solid Cancers
Source: PLoS Genet. 2008 Jul 18;4(7):e1000129. doi: 10.1371/journal.pgen.1000129 (PMC2444049; doi:10.1371/journal.pgen.1000129)
Supplement: Table S5 — Multivariate analysis for the PGC in primary tumors. (0.06 MB DOC) [file pgen.1000129.s010.doc]

## Table S5. Multivariate analysis for the PGC in primary tumors

| **Data Set** | **p-value** | | **Hazard ratio** | | **(95% CI)** | | | |  |
| --- | --- | --- | --- | --- | --- | --- | --- | --- | --- |
| **Sotiriou_Breast** |  | |  | | Lower | | | Upper |  |
| PGC | **0.02** | | 0.15 | | 0.03 | | | 0.74 |  |
| grade | 0.15 | | 1.68 | | 0.83 | | | 3.39 |  |
| node | 0.31 | | 0.48 | | 0.11 | | | 2.01 |  |
| size | 0.13 | | 1.47 | | 0.89 | | | 2.44 |  |
| age | 0.85 | | 1.01 | | 0.93 | | | 1.09 |  |
|  |  | |  | |  | | |  |  |
| **Wang_Breast** |  | |  | |  | | |  |  |
| PGC | **0.01** | | 0.47 | | 0.27 | | | 0.80 |  |
| ER | 0.18 | | 0.70 | | 0.41 | | | 1.18 |  |
|  |  | |  | |  | | |  |  |
| **Bild_Lung** |  | |  | |  | | |  |  |
| PGC | 0.66 | | 0.92 | | 0.62 | | | 1.35 |  |
| Stage | **0.03** | |  | |  | | |  |  |
| 1 | 0.84 | | 1.16 | | 0.27 | | | 5.01 |  |
| 2 | 0.93 | | 1.07 | | 0.21 | | | 5.40 |  |
| 3 | 0.09 | | 3.69 | | 0.82 | | | 16.58 |  |
|  |  | |  | |  | | |  |  |
| **Bild_Ovarian** |  | |  | |  | | |  |  |
| PGC | 0.07 | | 0.65 | | 0.41 | | | 1.04 |  |
| Stage (III vs. IV) | **0.04** | | 0.52 | | 0.27 | | | 0.97 |  |
|  |  | |  | |  | | |  |  |
| **Phillips_Glioma** | |  | |  | |  |  | | |
| PGC | | 0.09 | | 0.52 | | 0.24 | 1.11 | | |
| age | | 0.52 | | 1.01 | | 0.98 | 1.04 | | |
| Stage | | 0.09 | | 0.50 | | 0.22 | 1.13 | | |
| Gender | | 0.92 | | 0.97 | | 0.55 | 1.71 | | |
|  | |  | |  | |  |  | | |
| **Aronow_Colon** | |  | |  | |  |  | | |
| PGC | | **0.007** | | 0.20 | | 0.06 | 0.65 | | |
| M | | 0.13 | | 9.02 | | 0.54 | 151.66 | | |
| N | | 0.34 | | 1.89 | | 0.51 | 7.07 | | |
| T | | 0.11 | | 2.84 | | 0.78 | 10.36 | | |
| DUKESTAGE**^a^** | | 0.92 | | 0.00 | | 0.00 | 2.15E+52 | | |
| size | | 0.73 | | 0.95 | | 0.73 | 1.25 | | |
| age | | 0.27 | | 0.97 | | 0.92 | 1.02 | | |
| AJCCSTAGE | | 0.93 | | 279.96 | | 0.00 | 3.80E+57 | | |
| Gender | | 0.74 | | 0.81 | | 0.24 | 2.78 | | |

Note: We performed multivariate analysis using Cox regression (SPSS) to determine if the prognostic ability of the PGC was independent or associated with other known clinical variables. PGC expression behaved as an independent prognostic factor for survival in the two breast cancer data sets (Wang and Sotiriou) and colon data set when compared to other important determinants of patient survival such as estrogen receptor (ER) status. PGC expression was not an independent prognostic factor in the lung, ovarian and glioma cancer, and was correlated with tumor stage.
