# Supplementary material for: A Precisely Regulated Gene Expression Cassette Potently Modulates Metastasis and Survival in Multiple Solid Cancers
Source: PLoS Genet. 2008 Jul 18;4(7):e1000129. doi: 10.1371/journal.pgen.1000129 (PMC2444049; doi:10.1371/journal.pgen.1000129)
Supplement: Table S6 — Association between PGC genes and Myc/p53 genome binding loci. (0.03 MB DOC) [file pgen.1000129.s011.doc]

**Table S6. Association between PGC Genes and Myc/p53 Genome Binding loci**

|  | Myc^a^ | | | | P53^b^ | |
| --- | --- | --- | --- | --- | --- | --- |
|  | Myc-Induced | | Control | |
| TF-binding genes  in 5729-gene set | 390^c^ | | 227 | | 144 | |
| PGC | **0.042** | 7^d^ | 0.296 | 3 | 1 | 0 |

^a^ c-myc genomic binding sites identified by ChIP-PET in a human B cell line p493 expressing either high (turomrigenic) or low levels (physiological) of c-Myc under the control of a tetracycline-repressible promoter (Zeller et al., 2006).

^b^ P53 genomic binding loci in HCT116 after 5-fluorouracil stimulation (Wei et al., 2006).

PET clusters of size >=3 were used for analysis. P-values were calculated by hypergeometric distribution test and the significant values (p<0.05) are marked in bold.

^c^ Number of genes with Myc or P53 binding loci in the 5,729-gene set

^d^ Number of genes with Myc or P53 binding loci in the PGC signatures
