# Supplementary material for: A Precisely Regulated Gene Expression Cassette Potently Modulates Metastasis and Survival in Multiple Solid Cancers
Source: PLoS Genet. 2008 Jul 18;4(7):e1000129. doi: 10.1371/journal.pgen.1000129 (PMC2444049; doi:10.1371/journal.pgen.1000129)
Supplement: Text S1 — Supplementary methods. (0.09 MB DOC) [file pgen.1000129.s012.doc]

**Supplementary Methods**

Processing and Storage of Patient Tissue Samples

Primary human tumor and adjacent normal tissues were obtained from the Tissue Repository of the National Cancer Centre of Singapore (NCCS). Appropriate institutional approvals were obtained from the NCCS Tissue Repository and Ethics Committees. In the operating theater, morphologically visible tumor and adjacent matched normal tissues were removed by surgery and examined by a surgical pathologist to confirm the presence of cancer cells by cryosections. The tissue samples were divided into discrete aliquots, flash frozen and subsequently stored in liquid nitrogen. The training set used in this study comprises 341 samples (270 tumors and 71 adjacent normals) from patients with breast, colon, liver, lung, oesophagal and thyroid cancer (Tissue Type/Tumor/Normal : Lung/18/12, Thyroid/35/16, Liver/9/8, Oesophagus/16/13, Colon/9/9, Breast/183/13) For the independent in-house test set, we used another 99 tumor samples from patients with gastric or nasopharyngeal carcinoma (Gastric/53 tumors, NPC/46 tumors).

Permutation Testing and Repeated Random Sampling (RSS)
*Permutation Testing :* To test the association of the PGC genes with the tumor vs normal class distinction, we randomly shuffled the class labels (ie, tumor or non-malignant) of the training set samples to a series of randomized ‘tumor’ and ‘control’ sets. The permuted tumor and normal sets were assembled at proportions similar to the actual training data (ie 270 and 71 samples). The randomized training data was then used to re-identify permuted PGC signatures (pPGC), and this operation was repeated 1,000 times. The absolute numbers of genes in the pPGC were then compared with the numbers of genes in the true PGC identified using the actual training data. We also determined the frequency at which genes in the true PGC were re-identified in the pPGC signatures.

*Repeated Random Sampling test* *(RRS):* A RSS cross-validation procedure was used to evaluate the robustness of the PGC signature in different training sets (Michiels et al., 2005). We randomly divided the original training set 1,000 times into two parts: 1) a ‘training set’ comprising two thirds of the original set; and 2) a ‘test set’ comprising the remaining one third. The proportions of tumor and normal samples in both the RSS training and test sets were controlled to be similar to the original complete training set (ie, 270 tumors to 71 normals). For each of the 1,000 RSS training sets, we identified new PGC signatures (rPGC). Following the guidelines of Michels et al., 2005, we chose genes that were repeatedly selected in more than half of the 1,000 new rPGC signatures.

We also tested if the RSS signatures (rPGC) were also tightly regulated in the RSS test sets (tumor or control). First, we identified genes exhibiting similar restricted expression variation in the test data set, using the CV_T_ threshold cut-off (CV(Test) < CV_T_). Second, we determined the overlap between the RSS gene signatures rPGC (from training data) and the population of genes that were tightly regulated in the RSS test set. The hypergeometric distribution test was used to calculate the significance of the overlap. Significance was defined as p-value (p<0.01). There are two possible comparisons: (RSS Training -> RSS Test) rPGC 🡪rTumor_test_ and rPGC🡪rNormal_test_. If the PGC signatures are robust and phenotype-dependent, we would expect a significant enrichment for rPGC 🡪rTumor_test_, but not for rPGC🡪rNormal_test_. That is, for example, rPGC genes exhibiting restricted expression variations in tumors should not exhibit the same degree of tight regulation in normal tissues.

Selecting Metastatic Variants of HCT116 Cells

The *in vivo* selection procedure used to generate metastatic variants from the HCT116 colon cancer cell line has been previously described (Morikawa et al., 1988). Briefly, five-week-old female athymic mice were purchased from Animal Resources Centre (Canning Vale, WA, Australia) and maintained under specific pathogen-free conditions. 2x10^6^ cells (HCT116 or its variants) were suspended in 0.05 ml of PBS was injected into the medial spleen tip. Six to eight weeks after injection, the animals were sacrificed and post-mortem examination performed. The hepatic metastatic nodules were obtained, washed in McCoy’s 5A Modified medium containing 10% FBS, 100 U/ml penicillin, 100 μg/ml streptomycin and 0.25 μg/ml amphotericin B, and minced. Following incubation with 50 U/ml dispase I for 30 - 60 min at 37^o^C, the dispersed cells were filtered through a 100 μm filter. The cells obtained from the hepatic metastatic nodules after the first passage was named M1, and the *in vivo* selection procedure was repeated twice to obtain the M2 and M3 cell lines. Three independent replicates were profiled for each cell line. The HCT116 derived cell lines were characterized for their metastatic ability by injecting 1x10^6^cells into the spleen of the nude mice. The mice were sacrificed after 8 weeks and the number of liver metastases determined by visual inspection. The animal work performed was approved by the National University of Singapore Institutional Animal Care and Use Committee (NUS IACUC).

Metastasis Vector and GSEA analysis

To determine if PGC expression was correlated with the metastatic capacity of these cancer cell lines, we quantified the metastatic ability of HCT116 and its derivative cell lines. We assigned a numerical vector [1 3 7 10] to [HCT116 M1 M2 M3] based on the *in vivo* data. We then calculated the correlation between the metastasis vector and the expression values of the PGC. Gene Set Enrichment Analysis (GSEA), a modification of the weighted Kolmogorov-Smirnov (KS) statistic that provides a general statistical framework to test for the enrichment of gene expression profiles (Subramanian et al., 2005), was used to calculate the significance of the correlation. GSEA considers all the PGC genes and determines if these genes are enriched at the top (or bottom) of a list of markers ranked by the degree of correlation with a specific phenotype (ie, the metastasis vector). To ensure the robustness of this correlation, we also modified the metastasis vector to generate a series of derivative vectors with different scales: [1 2-4 7-9 10]. The correlation analysis was performed on each vector, and similar results were obtained (Data not shown).

Data analysis for Sotirioi_Breast data set

The Sotirioi_Breast set (breast cancer, GSE2990) includes 189 invasive breast carcinomas collected from two research institutions: John Radcliffe Hospital (Oxford, UK) and Uppsala University Hospital (Uppsala, Sweden). The CEL files were analyzed separately in four groups, according to the institutions and treatment status (ie, with or without systematic treatment) (Sotiriou et al., 2006). Clustering analysis was performed within each group. The low-level and high-level TSS tumors were then pooled together for survival analysis. The Kaplan-Meier test was performed on all the patients (Figure 5C, p=0.003), as well as on two populations with different treatment status. In both cases, higher levels of TSS expression were observed to associate with poor prognosis. The p-value is 0.049 for KJ125 (patients who did not receive systemic treatment; A) and 0.008 for KJX64 (patients who were treated with tamoxifen; B).
